# Fast and robust visual inspection of the coronary arteries based on live path tracking

**DOI:** 10.1186/1532-429X-11-S1-P290

**Published:** 2009-01-28

**Authors:** Javier Olivan Bescos, Jeroen Sonnemans, Marcel Breeuwer

**Affiliations:** Philips Healthcare, Best, Netherlands

**Keywords:** Maximum Intensity Projection, Volume Rendering, Path Tracking, Local Path, Optimal Visualization

## Introduction

A multitude of visualization techniques are available to inspect the coronary arteries in Steady-State Free-Precession (SSFP) MRI cardiac data, both 2D (planar, curved, and straightened reformatting) and 3D (volume rendering and maximum intensity projection). The workflow required to generate the visualizations includes user interactions such as segmentation, path tracking, and geometry manipulation (rotating, zooming, panning, etc). When these interactions are performed manually, the workflow becomes time consuming, and the inter-user reproducibility is low.

## Purpose

We describe a method that optimizes the workflow of local visual inspection of the coronary arteries by reducing the user interaction to a single mouse click. No a priori segmentation or path tracking is required, and the optimal position, orientation, and zoom factor of the views are found automatically, thereby increasing the efficiency and reducing and inter-user variability.

## Methods

**Figure 1:**
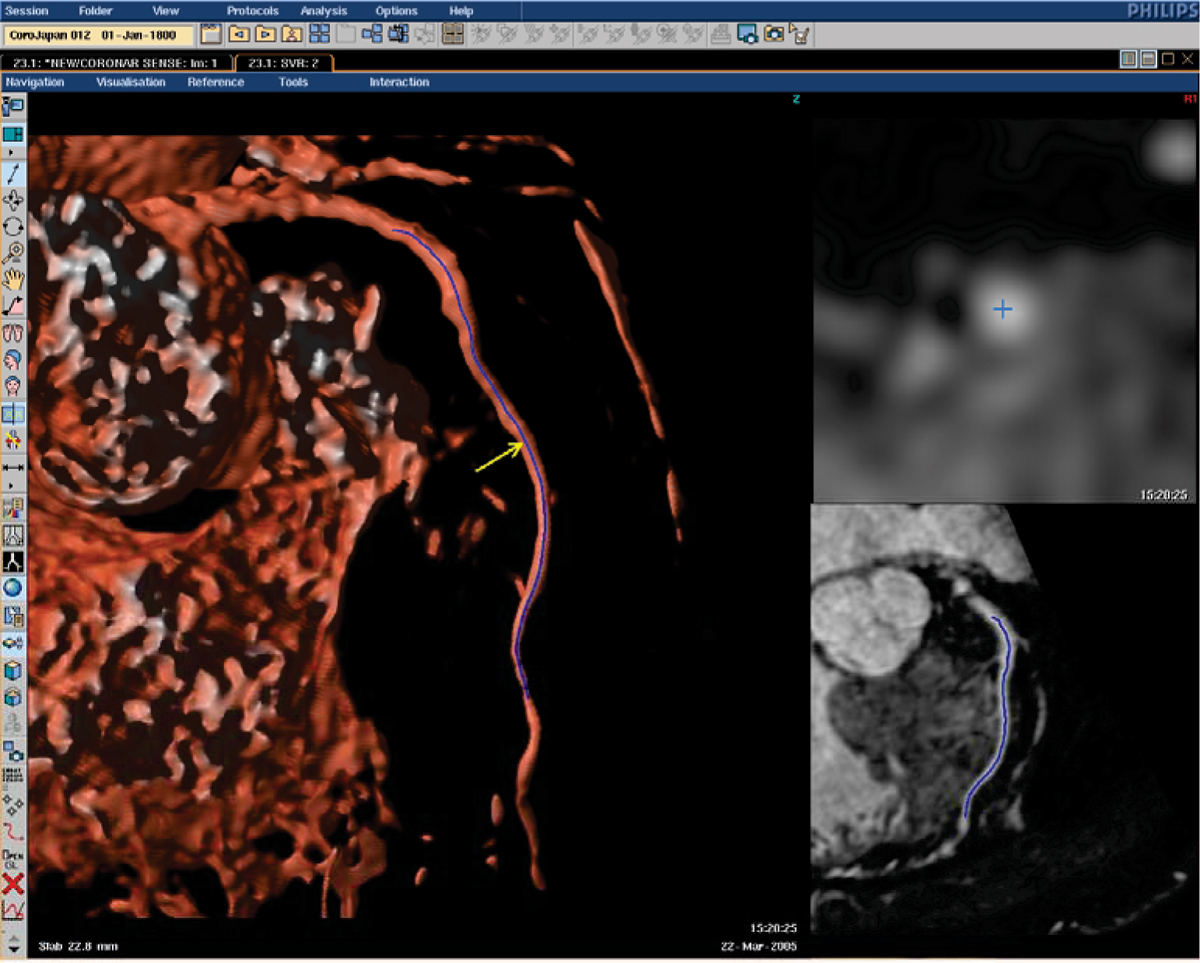
**The whole image was generated from a single mouse click (yellow arrow)**. Based on the local center line (in blue), a slab volume rendering *(left)*, a cross section *(top right)*, and a curvilinear reformat *(bottom right)* images were generated.

The method presented here should be considered as an advanced magnifying glass applied to the coronaries. When the user needs to inspect a particular location at one of the coronaries, a single mouse click locally computes a vessel centerline at the cursor position (yellow arrow in Figure 1). The length of the centerline can be modified with the mouse wheel at any time. A cross section and curvilinear reformatted image and a slab volume rendering aligned with the vessel are automatically generated from the local path (Figure 1). When necessary, the user can easily navigate along the selected vessel by hovering the mouse along the vessel in any of the views or by pressing the up/down keys. During navigation, the user may select different vessels with a single mouse click at any time.

## Results

The accuracy of the local path tracking has been evaluated on 11 data sets used in the work presented in [1]. A local centerline of 5 cm was computed with the local path tracking algorithm at the middle point of each golden standard path. The local path was compared to the golden standard using the Repeated Averaging Algorithm [2]. The mean error was 0.32 mm, with a standard deviation of 0.08 mm.

Our path tracking technique is very fast, it takes only tenths of a second including the generation of the visualizations. The user thus receives live feedback about the local coronary anatomy. The fact that all visualizations are automatically properly aligned decreases the inter-user variability associated with manual interactions. When different users click at about the same location, the same renderings will be generated.

## Conclusion

The method presented in this article greatly improves the workflow required to inspect the coronaries locally, while at the same time reducing the inter-user variability. A single mouse click generates optimal visualizations of the local geometry of the coronary vessels.
